# Synergistic antiproliferative effects of an mTOR inhibitor (rad001) plus gemcitabine on cholangiocarcinoma by decreasing choline kinase activity

**DOI:** 10.1242/dmm.033050

**Published:** 2018-05-14

**Authors:** Gigin Lin, Kun-Ju Lin, Frank Wang, Tse-Ching Chen, Tzu-Chen Yen, Ta-Sen Yeh

**Affiliations:** 1 Department of Medical Imaging and Intervention, Imaging Core Lab, Institute for Radiological Research, Clinical Metabolomics Core Lab, Chang Gung Memorial Hospital at Linkou, Chang Gung University, Taoyuan 333, Taiwan; 2 Department of Nuclear Medicine and Molecular Imaging Center, Chang Gung Memorial Hospital at Linkou, Chang Gung University, Taoyuan 333, Taiwan; 3 Department of Pathology, Chang Gung Memorial Hospital at Linkou, Chang Gung University, Taoyuan 333, Taiwan; 4 Department of Surgery, Chang Gung Memorial Hospital at Linkou, Chang Gung University, Taoyuan 333, Taiwan

**Keywords:** Cholangiocarcinoma, Choline kinase, FAS, Gemcitabine, Rad001

## Abstract

Although gemcitabine plus cisplatin is the gold standard chemotherapy regimen for advanced cholangiocarcinoma, the response rate has been disappointing. This study aims to investigate a novel therapeutic regimen [gemcitabine plus everolimus (rad001), an mTOR inhibitor] for cholangiocarcinoma. Gemcitabine, oxaliplatin, cetuximab and rad001 in various combinations were first evaluated *in vitro* using six cholangiocarcinoma cell lines. *In vivo* therapeutic efficacies of gemcitabine and rad001 alone and their combination were further evaluated using a xenograft mouse model and a chemically induced orthotopic cholangiocarcinoma rat model. In the *in vitro* study, gemcitabine plus rad001 exerted a synergistic therapeutic effect on the cholangiocarcinoma cells, irrespective of the *KRAS* mutation status. In the xenograft study, gemcitabine plus rad001 showed the best therapeutic effect on tumor volume change, and was associated with increased caspase-3 expression, decreased eIF4E expression, as well as overexpression of both death receptor- and mitochondrial apoptotic pathway-related genes. In a chemically induced cholangiocarcinoma-afflicted rat model, the gemcitabine plus rad001 treatment suppressed tumor glycolysis as measured by ^18^F-fludeoxyglucose micro-positron emission tomography. Also, increased intratumoral free choline, decreased glycerophosphocholine and nearly undetectable phosphocholine levels were demonstrated by proton nuclear magnetic resonance, supported by results of decreased choline kinase expression in western blotting. We concluded that gemcitabine plus rad001 has a synergistic antiproliferative effect on cholangiocarcinoma, irrespective of the *KRAS* mutation status. The antitumor effect is associated with activation of both death receptor and mitochondrial pathways, as well as the downregulation of choline kinase activity, resulting in a characteristic change in choline metabolism.

## Abstract (precis)

**Summary:** Rad001 plus gemcitabine exerts a synergistic antitumor effect on cholangiocarcinoma irrespective of *KRAS* mutation status, with underlying mechanisms involving activation of the death receptor, mitochondrial pathways and downregulated choline kinase activity.

## INTRODUCTION

Cholangiocarcinoma is a malignant biliary tract tumor that carries a very poor prognosis (de Groen et al., 1999; Khan et al., 2005). Although uncommon worldwide, its incidence reaches 87 per 100,000 in endemic areas, including Israel, Japan and South Eastern Asian countries such as Taiwan (Rajagopalan et al., 2004; Yeh et al., 2005). Surgical resection is the only treatment modality that offers a potential cure. Unfortunately, resection rates are low at the initial diagnosis because of early intrahepatic spread, nodal involvement, vascular invasion and distant metastasis. Other therapeutic strategies, including chemotherapy, radiation therapy, transplantation and photodynamic therapy, have been developed, but none has shown significant survival benefit in patients with advanced cholangiocarcinoma (de Groen et al., 1999; Khan et al., 2005). Gemcitabine, with or without platinum compounds, is currently the most effective chemotherapy regimen for advanced biliary tract cancer, although the response rate is only ∼20% (André et al., 2004; Eckel and Schmid, 2007; Valle et al., 2010, 2009). In addition, epidermal growth factor receptor (EGFR) is activated by bile acids and has a role in the carcinogenesis of cholangiocarcinoma through the induction of cyclooxygenase-2 expression via an MAPK protein cascade. Accordingly, neutralizing antibodies that target the extracellular domain of EGFR (e.g. cetuximab) have sporadically shown clinical efficacy in advanced cholangiocarcinoma (Paule et al., 2007).

Mammalian target of rapamycin (mTOR) is a conserved serine/threonine kinase that regulates cell growth and metabolism. The mTOR pathway is dysregulated in various cancers including cholangiocarcinoma (Chung et al., 2009; Ma and Blenis, 2009), making mTOR an important target for the development of new anticancer drugs (Faivre et al., 2006). Everolimus (rad001), a 40-O-(2-hydroxyethyl) derivative of rapamycin, conjugates with FK binding protein-12 (FKBP-12; FKBP1A) to inhibit the activation of mTOR. Although rad001 exhibits antitumor activity both as a single oral agent and in combination with other anticancer agents (Mabuchi et al., 2007; Majumder et al., 2004; Mondesire et al., 2004; Motzer et al., 2008; Piguet et al., 2008; Yan et al., 2006; Zhang et al., 2009), its therapeutic effect on cholangiocarcinoma has yet to be clarified.

Phosphocholine (PC) is both a precursor and a breakdown product of phosphatidylcholine, the most abundant phospholipid in the biological membranes. Increased levels of PC and total choline, as detected by ^1^H or ^31^P magnetic resonance spectroscopy (MRS), are characteristics of cancer cells, including breast, prostate, colon, lung, ovarian and brain tumors (Ackerstaff et al., 2003; Iorio et al., 2005; Venkatesh et al., 2012). The significance of these studies is that specific changes in the levels of the phospholipid precursors and catabolites in the context of solid cancers might be a useful biochemical indicator of tumor progression and/or treatment response.

This study aimed to evaluate the therapeutic effects of rad001 and gemcitabine on cholangiocarcinoma, and elucidate the molecular and metabolic mechanisms underlying their antitumor activity.

## RESULTS

### Selection of optimal drug regimens for cholangiocarcinoma cells

**Fig. 1.**
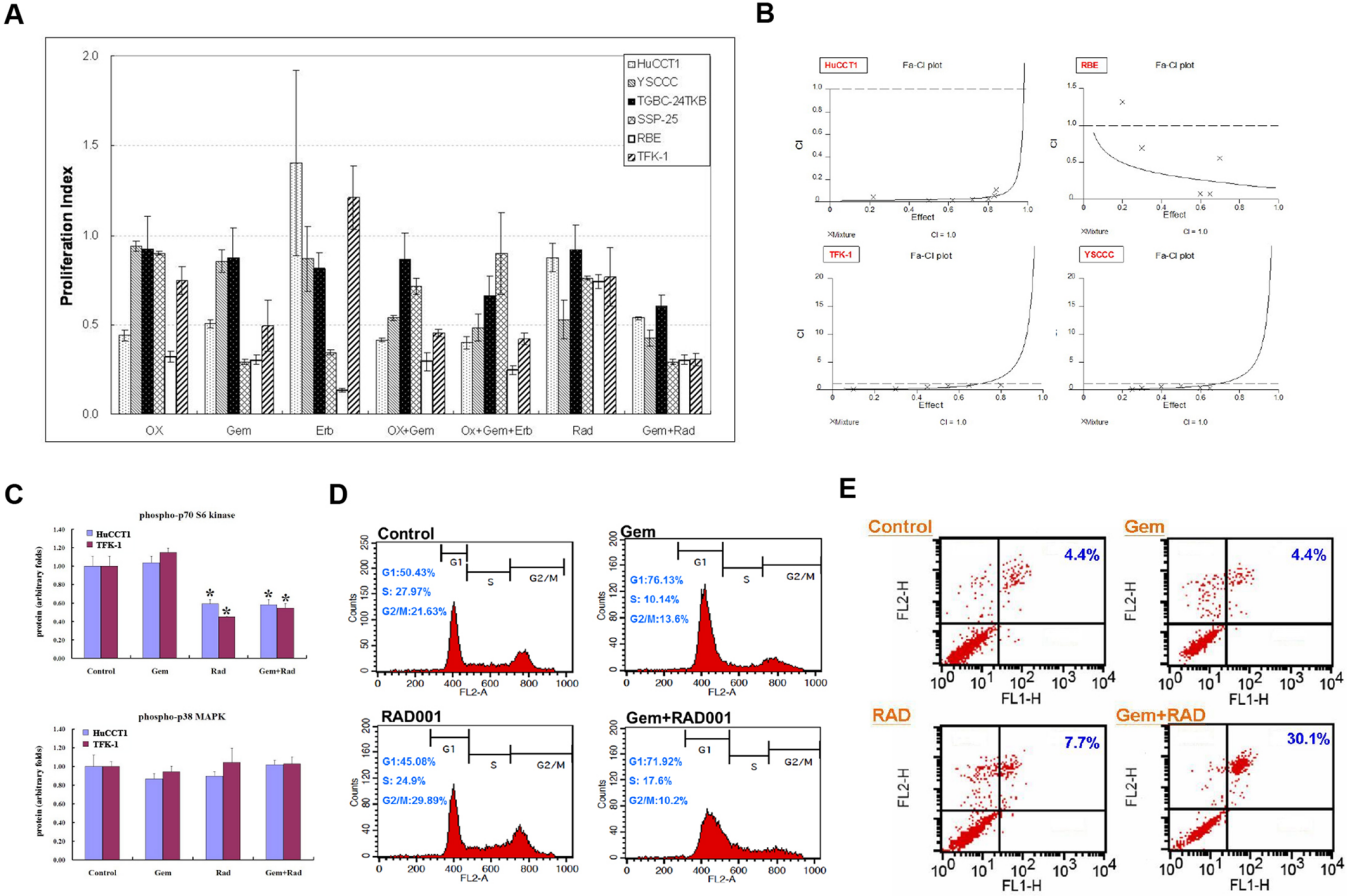
**Optimization of mTOR inhibition for treatment of cholangiocarcinoma.** (A) Proliferation index of six cholangiocarcinoma cell lines treated with different regimens (*n*=4 in each group). We examined the corresponding proliferation indices based on the HuCCT1 cell line and applied the dosage to the other five cholangiocarcinoma cell lines treated with mono-therapy and combined therapy, respectively. OX, oxaliplatin; Gem, gemcitabine; Erb, cetuximab; Rad, rad001. (B) The interaction between rad001 and gemcitabine in cholangiocarcinoma cell lines was determined by median effects analysis to derive a combination index (CI). (C) Phosphorylation of p70S6K and p38MAPK in HuCCT1 and TFK-1 cells detected by the Bio-Plex phosphoprotein assay (*n*=4 in each group). *P<0.05 versus control group. (D) Cell cycle changes in HuCCT1 cells detected by flow cytometry (*n*=4 in each group). (E) Percentage of apoptotic HuCCT1 cells detected by an Annexin V-FITC Apoptosis Kit (*n*=4 in each group). Error bars indicate s.d.

The corresponding proliferation indices of the six cholangiocarcinoma cell lines treated with standard IC_50_ dosages of various therapeutic regimens are shown in Fig. 1A. Gemcitabine exhibited superior therapeutic efficacy against all cholangiocarcinoma cell lines as a single-agent therapy, compared with oxaliplatin, cetuximab and rad001. Gemcitabine plus rad001 was the most promising combination therapeutic regimen, in comparison with gemcitabine plus oxaliplatin and gemcitabine plus oxaliplatin plus cetuximab. The median effects analysis demonstrated that rad001 and gemcitabine had a synergistic effect on both *KRAS* mutation (HuCCT1, RBE) and wild-type (TFK-1, YSCCC) cell lines with a combination index (CI)<1 (Fig. 1B).

### Rationale of mTOR inhibition for treatment of cholangiocarcinoma

To assess the pharmacological effects of rad001 and gemcitabine, four treatment groups were assigned for *in vitro* and *in vivo* experiments: control, gemcitabine-treated (Gem), rad001-treated (Rad) and gemcitabine plus rad001-treated (Gem+Rad) groups. In the *in vitro* analyses, phosphorylation of p70S6K (RPS6KB1), but not p38MAPK (MAPK14), was significantly decreased in the Rad and Gem+Rad groups in both HuCCT1and TFK-1 cells, indicating that Gem and Gem+Rad specifically inhibited the downstream mTOR signaling irrespective of the *KRAS* mutation status. Gem might not synergistically enhance the effect of Rad to inhibit mTOR (Fig. 1C). Regarding cell cycling, a smaller percentage of HuCCT1 cells entered the G2/M phase in the Gem+Rad group compared with the Gem group (10.2% versus 13.6%, *P*<0.05; Fig. 1D, Table 1). Furthermore, the percentage of apoptotic HuCCT1 cells in the Gem+Rad group was significantly increased compared with the Gem group (30.1% versus 4.4%, *P*<0.01; Fig. 1E).

**Table 1.**
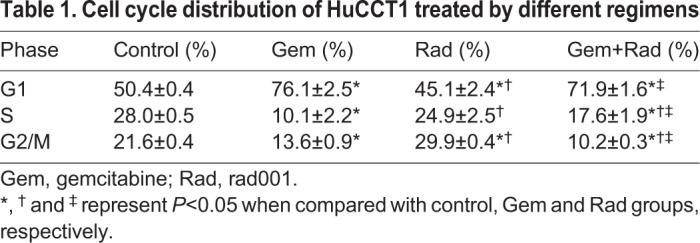
**Cell cycle distribution of HuCCT1 treated by different regimens**

### Evaluation of therapeutic response using a xenograft model

**Fig. 2.**
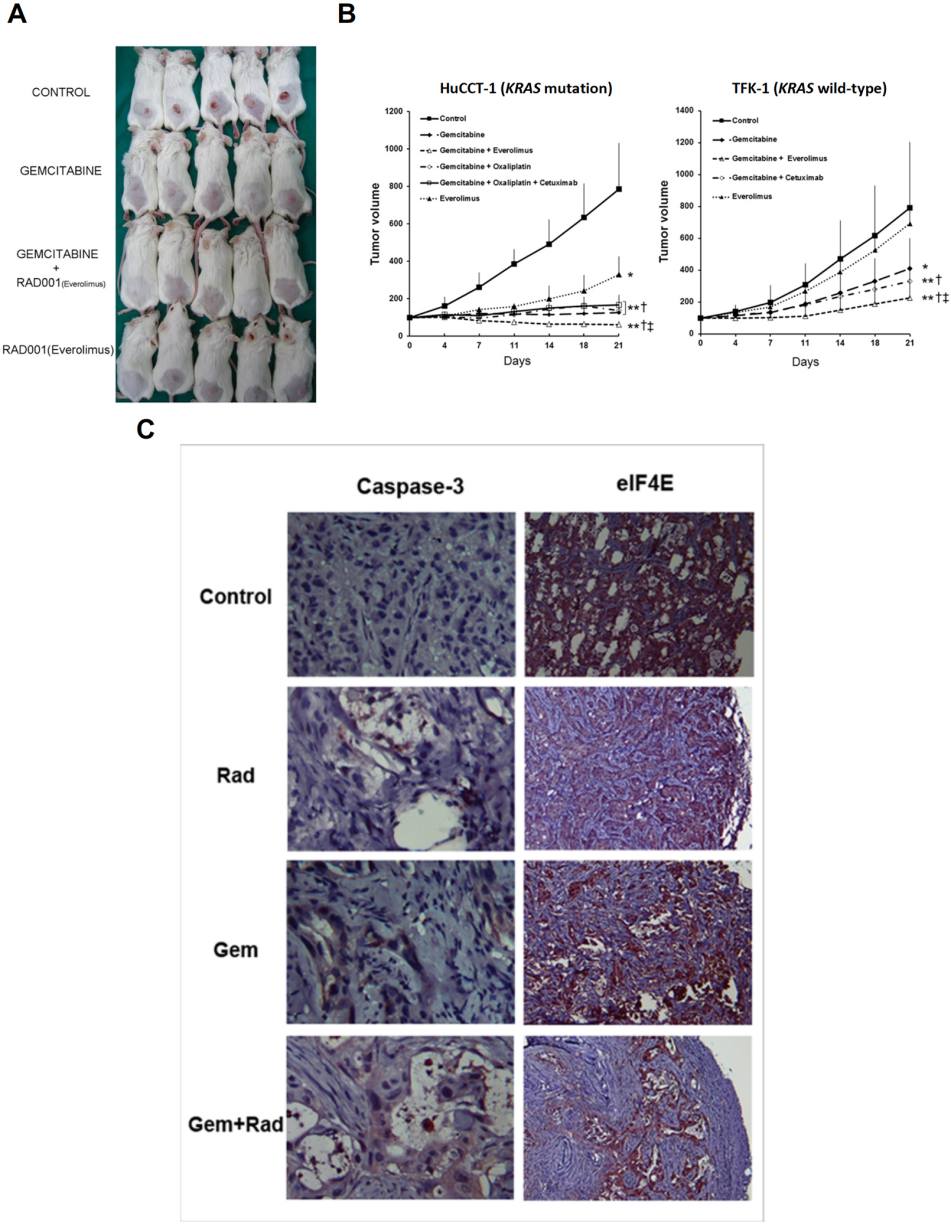
**Therapeutic response of HcCCT1 and TFK-1 xenograft models.** (A) Representative features of tumor growth in HuCCT1 xenograft models after a 3-week treatment (*n*=8 in each group). (B) Left: quantitative analysis of tumor growth in HuCCT1 xenograft models after a 3-week treatment. *P<0.05 and **P<0.01 versus control group, respectively; ^†^*P*<0.05 versus Rad group; ^‡^*P*<0.05 versus Gem group. Right: quantitative analysis of tumor growth in TFK-1 xenograft models after a 3-week treatment (*n*=8 in each group). *P<0.05 and **P<0.01 versus control group, respectively; ^†^*P*<0.05 versus Gem group; ^‡^*P*<0.05 versus Gem+cetuximab group. (C) Representative expression of caspase-3 and eIF4E in HuCCT1 xenograft model detected by immunohistochemistry (*n*=8 in each group). Original magnification, 400× for caspase -3 and 40× for eIF4E. Error bars indicate s.d.

Xenograft experiments were conducted to evaluate the measurable volumetric changes of the cholangiocarcinomas treated with various drug regimens. The representative therapeutic response of the HuCCT1 xenograft model after various treatments for 3 weeks is shown in Fig. 2A. The therapeutic responses of HuCCT1 and TFK-1 xenograft models treated for 3 weeks were analyzed (Fig. 2B). Gem+Rad regime conferred the most promising therapeutic response in both HuCCT1 and TFK-1 xenograft models. Representative images of caspase-3 and eIF4E expression in the HuCCT1 xenograft are shown in Fig. 2C. The expression of caspase-3 in the control, Gem, Rad and Gem+Rad groups was 2.1±0.7, 9.4±1.6, 5.1±1.1 and 14.5±2.3 per high-power field (HPF), respectively (*P*<0.01). In contrast, the expression of eIF4E in the control, Gem, Rad and Gem+Rad groups was 14.2±1.3, 8.9±1.5, 11.3±2.3 and 4.6±0.7 per HPF, respectively (*P*<0.01).

### Altered apoptotic signaling after gemcitabine and rad001 combination therapy

**Fig. 3.**
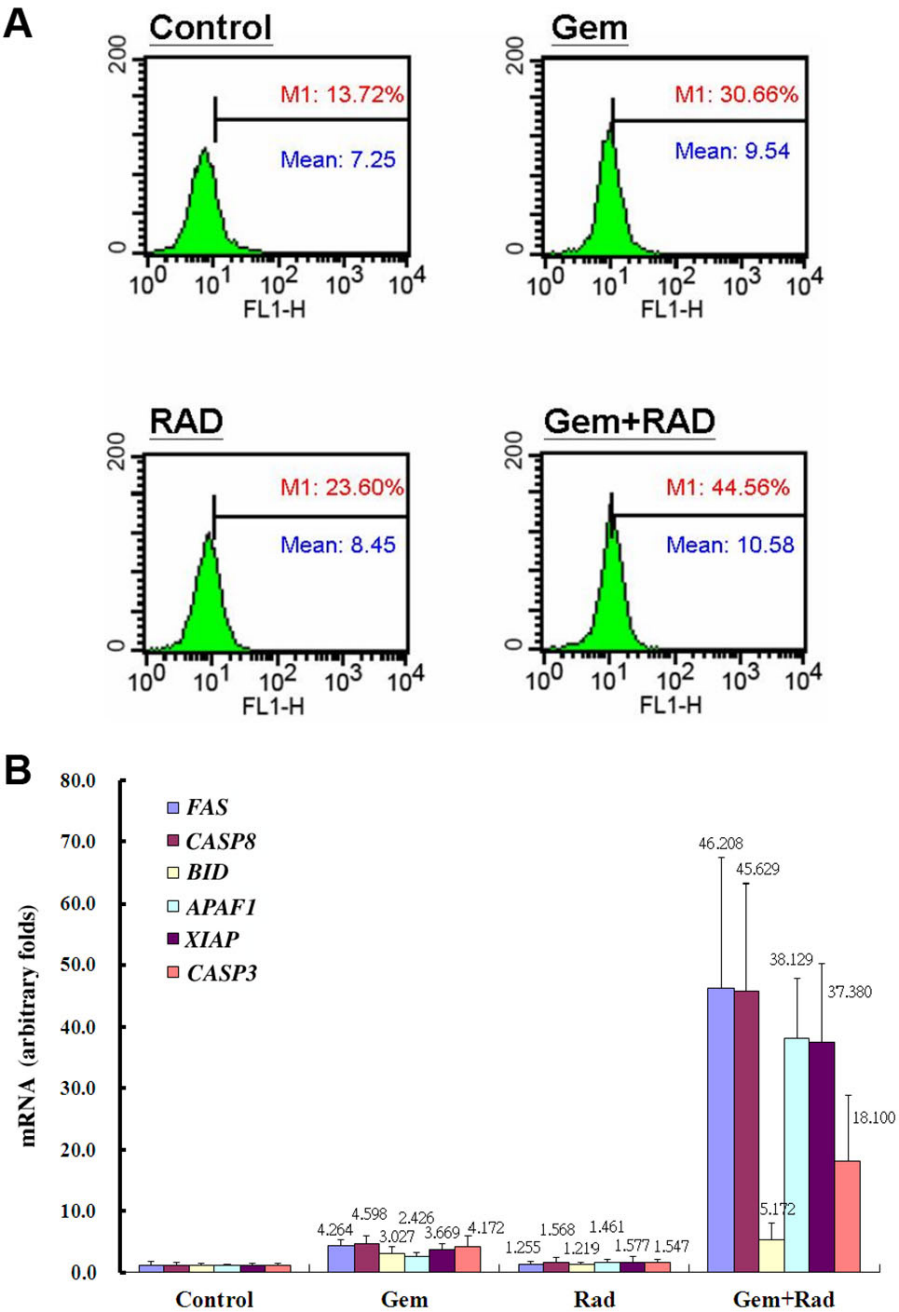
**Altered apoptotic signaling after gemcitabine and rad001 combination therapy.** (A) Percentages (red digits) and intensities (blue digits) of FAS-positive HuCCT1 cells in control, gemcitabine-treated, rad001-treated, and gemcitabine plus rad001-treated groups detected by flow cytometry (*n*=4 in each group). (B) The expression of *FAS*, *CASP8*, *BID*, *APAF1*, *XIAP* and *CASP3* mRNA in the HuCCT1 xenograft model detected by quantitative real-time PCR (*n*=6 in each group). *P<0.05 and **P<0.01 versus control group, respectively. ^†^*P*<0.05 and ^††^*P*<0.01 versus Gem group, respectively; ^‡^*P*<0.01 versus Rad group. Error bars indicate s.d.

Based on the xenograft model, the percentages of FAS-positive HuCCT1 cells in the control, Gem, Rad and Gem+Rad groups, as detected by flow cytometry, were 13.7%, 30.6%, 23.6% and 44.5%, respectively (*P*<0.01), and the intensity of FAS-positive HuCCT1 cells was 7.2, 9.5, 8.4 and 10.5, respectively (*P*<0.01; Fig. 3A). The expression of six apoptotic genes, including *FAS*, *CASP8*, *BID*, *APAF1*, *XIAP* and *CASP3*, derived from the treated HuCCT1 xenograft using quantitative real-time polymerase chain reaction (qPCR), is shown in Fig. 3B. The expression of these six genes was modestly increased in the Gem and Rad groups compared with the control group, whereas the expression of all six genes was significantly increased in the Gem+Rad group compared with the other groups.

### Metabolic response of orthotopic cholangiocarcinoma rat model as detected by ^18^F-FDG microPET and ^1^H NMR

**Fig. 4.**
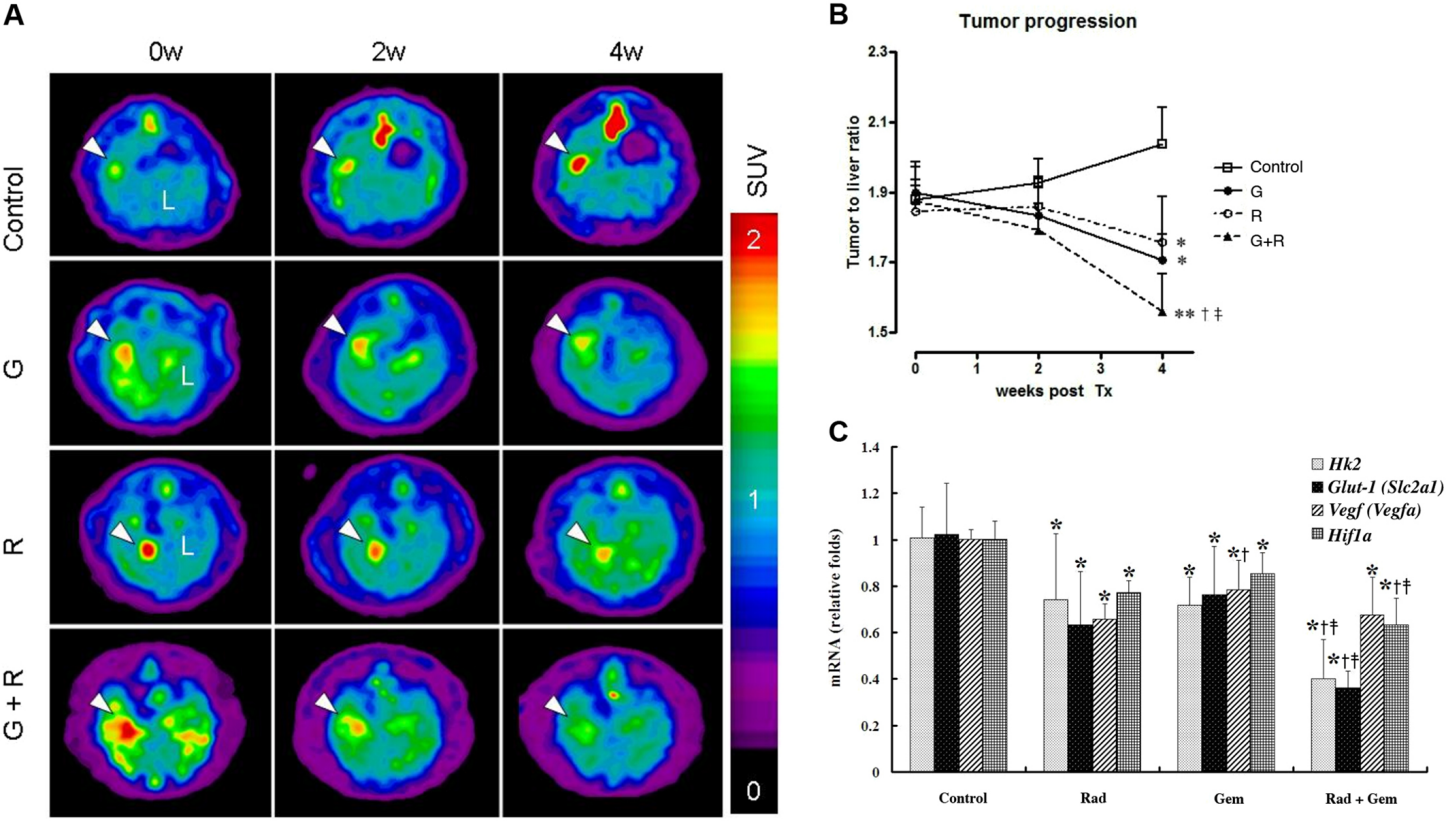
**Metabolic response of the orthotopic cholangiocarcinoma rat model, as detected by ^18^F-FDG microPET.** (A) Representative image of ^18^F-FDG microPET on cholangiocarcinoma-afflicted rats (*n*=6 in each group). (B) Metabolic response of cholangiocarcinoma-afflicted rats detected by ^18^F-FDG microPET was quantitatively analyzed (*n*=6 in each group). G, gemcitabine; R, Rad001. *P<0.05 and **P<0.01 versus control group, respectively; ^†^*P*<0.05 versus Rad group; ^‡^*P*<0.05 versus Gem group. (C) Expression of *Glut-1*, *Hk2*, *Hif1a* and *Vegf* mRNAs in rat cholangiocarcinoma detected by quantitative real-time PCR (*n*=6 in each group). *P<0.05 versus control group; ^†^*P*<0.05 versus Rad group; ^‡^*P*<0.05 versus Gem group. Error bars indicate s.d.

The representative *in vivo* metabolic response of cholangiocarcinoma-afflicted rats to various treatments as detected by ^18^F-fludeoxyglucose micro-positron emission tomography (^18^F-FDG microPET) is shown in Fig. 4A. The Gem+Rad group had the most significant metabolic response after two cycles of treatment (Fig. 4B). The expression of *Glut-1* (*Slc2a1*), *Hk2*, *Hif1a* and *Vegf* (*Vegfa*) mRNAs of the corresponding surgical specimens as detected by qPCR is shown in Fig. 4C. Of these, the expression of *Hk2* and *Glut-1* mRNA in the Gem+Rad group was reduced by half in comparison with the control group. The difference in the expression of these mRNAs correlated with the standardized uptake value (SUV) changes in FDG microPET. The tumor response in the cholangiocarcinoma-afflicted rats was confirmed by histopathological examination. Results derived from the histomorphological score and Ki67 (Mki67) labeling index of the corresponding surgical specimen also supported the findings in the FDG microPET study (Table 2).

**Table 2.**
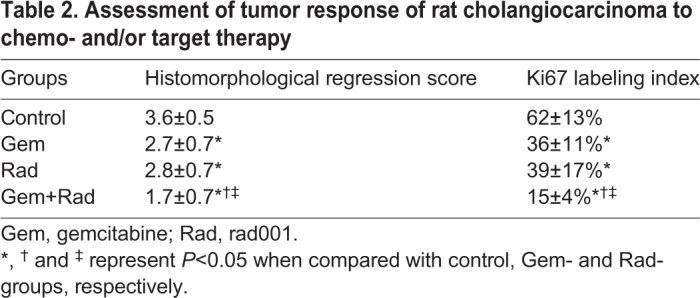
**Assessment of tumor response of rat cholangiocarcinoma to chemo- and/or target therapy**

**Fig. 5.**
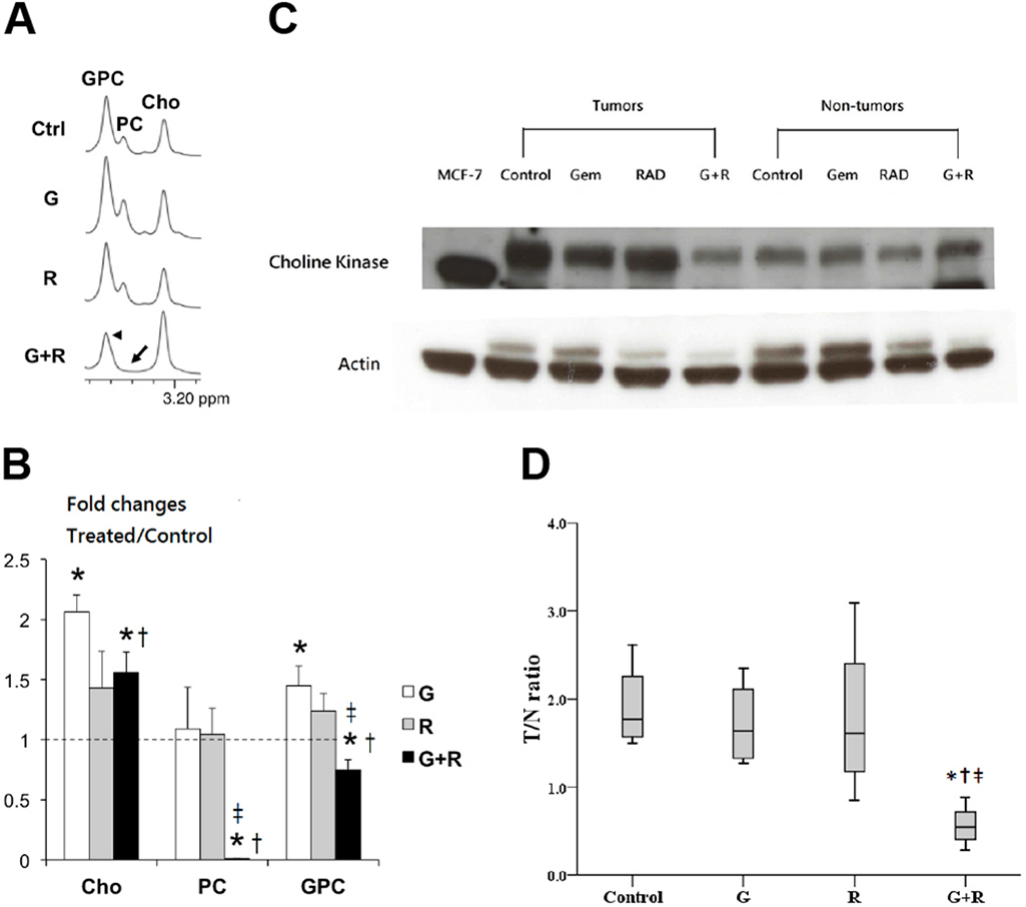
**Alteration of choline-associated metabolites in the treated cholangiocarcinoma-afflicted rats, as detected by ^1^H NMR.** (A) Representative NMR spectrum of choline-associated metabolites in cholangiocarcinoma-afflicted rats. Cho, choline; PC, phosphocholine; GPC, glycerophosphocholine. (B) Relative fold changes of intratumoral choline-associated metabolites after various treatment for 4 weeks (*n*=6 in each group). G, gemcitabine; R, rad001. *P<0.05 versus control group. (C) Representative features of choline kinase of treated cholangiocarcinoma-afflicted rats detected by western blotting. Gem, gemcitabine; Rad, rad001. MCF-7, a breast cancer cell line, served as a positive control; actin served as an internal control. (D) Quantitative analysis of CK in treated cholangiocarcinoma-afflicted rats detected by western blotting (*n*=6 in each group). G, gemcitabine; R, rad001. *P=0.021 versus control; ^†^*P*=0.021 versus G; ^‡^*P*=0.043 versus R. Error bars indicate s.d.

The alteration of choline-associated metabolites in the treated cholangiocarcinoma-afflicted rats was analyzed by ^1^H nuclear magnetic resonance (NMR), with a particular emphasis on the free choline, PC and glycerophosphocholine (GPC) levels (Fig. 5A). A significant increase in the free choline level of the Gem+Rad group was associated with dramatic disappearance of PC and a significantly reduced level of GPC (Fig. 5B). In contrast, the Gem group exhibited a significant increase in the free choline level without significant changes in PC and GPC levels. No significant change in the choline-associated metabolites was observed in the Rad group. Choline kinase (CK; CHKA), an enzyme that catalyzes the phosphorylation of free choline using ATP to produce PC and plays a rate-limiting regulatory role in the phosphatidylcholine biosynthesis, was assayed to determine the underlying mechanism that was responsible for the observed changes in the PC levels. Of the four treatment groups, the CK level detected by western blotting was lowest in the Gem+Rad group (Fig. 5C,D).

## DISCUSSION

The presence of the tyrosine kinase-AKT-mTOR axis in the carcinogenesis of cholangiocarcinoma (Chung et al., 2009) has provided us with an opportunity to evaluate the therapeutic role of an mTOR inhibitor, rad001, in the management of this disease. We have shown in this study that rad001 treatment resulted in reduction of expression of the downstream molecules of mTOR, such as p70S6K-1, but not p38MAPK. Furthermore, we demonstrated, for the first time, that gemcitabine plus rad001 exerted a synergistic antitumor effect on cholangiocarcinoma, independent of the *KRAS* mutation status. In contrast, the EGFR monoclonal antibody, cetuximab, had a modest therapeutic effect on the *KRAS* wild-type cholangiocarcinoma xenograft (TFK-1), but not the *KRAS* mutated xenograft (HuCCT1). This finding is consistent with previous clinical experience on colorectal and nonsmall cell lung cancers (NSCLCs) (Bardelli and Siena, 2010; Massarelli et al., 2007). The *in vivo* therapeutic effect of gemcitabine plus rad001 was macroscopically evaluated and confirmed by measuring changes in the tumor volume in the xenograft model, and assessing the metabolic response in the orthotopic rat cholangiocarcinoma model using ^18^F-FDG microPET. The downstream molecules of mTOR (Ki67, Hif1a, Hk2, Glut-1 and Vegf) were all attenuated in the Rad+Gem group. This observation reflected the changes in SUV identified in ^18^F-FDG microPET.

The available evidence in the literature suggests that gemcitabine primarily acts on an intrinsic (mitochondrial) apoptotic pathway in NSCLCs and pancreatic cancer to achieve its pharmacological effect. Gemcitabine-mediated activation of caspase-8 is a late and mitochondrial-dependent event which is independent of FAS-FASL (FASLG) signaling (Ferreira et al., 2000; Salvesen and Duckett, 2002). Que et al. reported that cholangiocarcinoma cells synthesized FASL and FAS and that FASL expressed by these cells induced host lymphocyte cell death (Que et al., 1999). Shimonishi et al. further demonstrated upregulation of FASL in the early stages and downregulation of FAS in the advanced stages of cholangiocarcinoma, the latter of which was responsible for evasion from host immunity and disease progression (Shimonishi et al., 2000). To understand the mechanism by which rad001 and gemcitabine acted synergistically to enhance apoptosis in the cholangiocarcinoma cells, we examined six key apoptotic genes that were involved in the extrinsic and intrinsic cell death pathways. These included *FAS*, *CASP8*, *BID*, *APAF1*, *XIAP* and *CASP3*. Among these six genes, the former two represent the death receptor (extrinsic) pathway, whereas the latter three represent the mitochondrial (intrinsic) pathway, with both pathways intermediated by BID (Salvesen and Duckett, 2002). Of particular importance, XIAP, an X-linked inhibitor of apoptosis, is thought to directly inhibit certain caspases such as caspase-3, caspase-7 and caspase-9. Shrikhande et al. showed that XIAP was overexpressed in pancreatic cancer and contributed to chemoresistance (Shrikhande et al., 2006). Our data revealed that both death receptor-related (*FAS* and *CASP8*) and mitochondrial pathway-related genes (*APAF1* and *CASP3*) in the Gem+Rad group were increased 10-fold in comparison with those of the Gem group, indicating that both death receptor and mitochondrial pathways were recruited and co-activated by the combination therapy. It is noteworthy that *XIAP* was simultaneously increased by up to 10-fold in the Gem+Rad group, along with the pro-apoptotic genes, in comparison with the Gem group. Our findings suggested that XIAP plays a major role in arresting the apoptotic process and potentially contributing to drug resistance.

Early detection of response to therapy is desirable but not easily achieved using conventional imaging techniques such as computed tomography or magnetic resonance imaging. Biological cancer treatment might be associated with stagnation of cellular proliferation or cellular clonal changes rather than obvious tumor shrinkage in the early stage of the therapeutic response. Cancer metabolism has emerged in recent studies as a powerful link between deregulated signaling pathways and altered cellular metabolism. Specific endogenous metabolites and metabolic fluxes within cells and tissues *in vivo* and *in vitro* can be readily identified using ^31^P, ^1^H or ^13^C MRS. Of them, derangement in the choline metabolism during carcinogenesis and treatment has been increasingly recognized in various solid cancers (Ackerstaff et al., 2003). CK catalyzes the phosphorylation of choline to yield PC in the biosynthesis of phosphatidylcholine, a process known as the Kennedy pathway (Ackerstaff et al., 2003; Ramírez de Molina et al., 2008). The enzyme is modulated by RAS and interacts with HIF1A (Ramírez de Molina et al., 2002a). It might also play a role in the cell cycle regulation (Gruber et al., 2012), MAPK and PI3K (PIK3CA)/AKT protein signaling pathways (Yalcin et al., 2010), as well as acting as a potential biomarker for various solid cancers (Ramírez de Molina et al., 2002b). Specific inhibitors against CK are to be anticipated soon in clinical trials as these agents have shown promising antitumor effects in preclinical studies (Ackerstaff et al., 2003; Al-Saffar et al., 2010; Ramírez de Molina et al., 2008, 2002a). In the present study, we have implemented ^1^H NMR to detect alterations in choline-associated metabolites in the treated cholangiocarcinoma-afflicted rats. Downregulated CK activity, presenting as an accumulation of free choline and nearly undetectable levels of PC in the Gem+Rad group, was confirmed by significantly decreased expression of CK in western blotting. Such downregulation of CK following mTOR inhibition was associated with HIF1A and PI3K/AKT/mTOR pathways, with the downregulation of *Hif1a* mRNA correlating with the respective CK levels in the Gem+Rad, Gem and control groups. In line with our study results, Glunde et al. demonstrated a similar process of CK regulation by HIF1A signaling in human prostate cancer (Glunde et al., 2008). Furthermore, using a PI3K inhibitor, PI-103, Al-Saffar et al. were able to demonstrate downregulation of CK levels in prostate and colon cancers, with a corresponding decrease in PC levels (Al-Saffar et al., 2010). Again, by silencing CK using small interfering RNA (siRNA), Yalcin et al. showed that PC was reduced through the PI3K/AKT/mTOR pathways, leading to the inhibition of cervical cancer cell growth (Yalcin et al., 2010).

The change in GPC levels after biological agent treatment is another interesting issue. Al-Saffar et al. reported that blockade of PI3K with LY294002 (a relatively less potent, nonselective inhibitor of PI3K) was associated with a decrease in PC levels as well as an elevation in GPC content in human breast cancer cells (Beloueche-Babari et al., 2006). In contrast, the authors noted that both PC and GPC levels were below the detection limit of MRS when the HCT116 colon cancer cells were treated instead with PI-103, a potent selective inhibitor of class 1 PI3K (Al-Saffar et al., 2010). Accordingly, they concluded that the contradictory response of the GPC levels following LY294002 and PI-103 treatments was probably related to other off-target effects of the former inhibitor. In our study, the characteristic profile of the choline metabolites in the cholangiocarcinoma cells treated by gemcitabine plus rad001 was most likely a result of a potent and selective targeting against the PI3K/AKT/mTOR signaling pathway. As there is an increase in choline and a decrease in its downstream metabolite, PC, we expect to observe an accumulation of choline signal on ^11^C-choline PET when the treatment is effective. A future *in vivo* imaging study using ^11^C-choline PET or magnetic resonance spectroscopy imaging would better facilitate longitudinal monitoring of the relevant biological response.

In conclusion, our preclinical study shows that gemcitabine plus rad001 exerts a synergistic antitumor effect on cholangiocarcinoma irrespective of the *KRAS* mutation status. The underlying mechanisms of antitumor activity are associated with activation of the death receptor and mitochondrial pathways, as well as the downregulation of CK activity, resulting in a specific change in choline metabolism.

## MATERIALS AND METHODS

### Cholangiocarcinoma cell lines

Six cholangiocarcinoma cell lines used in this study (HuCCT1, SSP-25, RBE, YSCCC, TGBC-24TKB and TFK-1) were purchased from RIKEN Cell Bank (Tsukuba, Japan). The HuCCT1, SSP-25 and RBE cell lines had a *KRAS* mutation, whereas the remaining three cell lines were *KRAS* wild type. All *in vitro* studies were performed at least in triplicate.

### *In vitro* drug cytotoxicity assay

Oxaliplatin (TTY Biopharm, Taiwan), gemcitabine (Gemmis, TTY Biopharm), cetuximab (Erbitux, Boehringer Ingelheim, Germany), and everolimus (rad001, Novartis, Switzerland), alone or in combination, were first used to treat the cholangiocarcinoma cell lines *in vitro*. The half-maximal inhibitory concentration (IC_50_) was determined using a cell proliferation reagent, WST-1 (Roche Applied Science, Germany).

### Analysis of interactions

A commercial software package (CalcuSyn, Biosoft, UK) was used to perform median effect analysis as described by Chou and Talalay (Chou and Talalay, 1984). The combination index (CI) was calculated using the formula: CI=(D)1/(Dx)_1_+(D)2/(Dx)_2_, where CI=1 indicated an additive effect, CI>1 an antagonistic effect, and CI<1 a synergistic effect.

### Flow cytometry

Both adherent and floating cholangiocarcinoma cells were collected 72 h after various drug treatments for cell cycle analysis. Cells were passed through a fluorescence activated cell sorter (FACS Caliber, BD Biosciences, USA), and data were acquired using CellQuest (BD Biosciences) software. The cell cycle was modeled using ModFit software (Venty Software, USA). The separation of cells into G0/G1, S and G2/M phases was based on linear fluorescence intensity after staining with propidium iodide. Cells were labeled with the Annexin V-FITC Apoptosis Kit (BD Biosciences-Clontech) and analyzed on a FACS Caliber to detect apoptosis. The cell surface expression of FAS after various drug treatments for 16 h was assessed. Indirect immunofluorescence staining for FAS was performed with an IgG1 mouse anti-FAS primary monoclonal antibody (DX2 clone, BD Biosciences) and a fluorescein isothiocyanate (FITC)-conjugated secondary antibody (goat anti-mouse Ig) (BD Biosciences). The percentage and intensity of FAS-positive cells were analyzed by the FACS Caliber.

### Bio-Plex phosphoprotein assay

Cholangiocarcinoma cells were treated with various drug regimens, and protein lysates were prepared using a cell lysis kit (Bio-Rad, USA). Phospho-p38MAPK and phospho-p70S6 kinase were detected by the Bio-Plex phosphoprotein and total protein assay kits (Bio-Rad) according to the manufacturer's protocols.

### Xenograft mice model

To initiate tumor xenograft, cholangiocarcinoma cells, HuCCT1 and TFK-1, were implanted subcutaneously (2×10^6^ cells) in the flank of the Fox Chase severe combined immunodeficiency (SCID) mice (BioLASCO, Taiwan). Six therapeutic regimens were provided, as follows: gemcitabine 200 mg/kg by intraperitoneal injection (I.P.)+oxaliplatin 5 mg/kg I.P. every week (GEMOX); gemcitabine 200 mg/kg I.P.+oxaliplatin 5 mg/kg I.P.+cetuximab 2 mg/kg I.P. every week (GEMOX+cetuximab); gemcitabine 200 mg/kg I.P. every week (GEM); rad001 5 mg/kg orally twice every week (Rad); gemcitabine, 200 mg/kg I.P. every week+rad001 5 mg/kg orally twice every week (GEM+Rad); and saline with the same volume I.P. every week (control group). Tumor size did not exceed 1000 mm^3^ (∼4% of mouse body weight) in compliance with animal welfare regulations. At the end of the third week, the xenografts were measured and retrieved. The surgical specimens were divided into two portions; half was kept frozen and stored at −80°C and the remaining half was prepared as a paraffin block. All animal experiments were performed according to a protocol approved by the Institutional Animal Care and Use Committee, Chang Gung University and Chang Gung Memorial Hospital, Taiwan (IACUC 2012092401). The care and use of experimental animals conformed with the regulatory standards.

### Immunohistochemical staining

Formalin-fixed, paraffin-embedded tissues were cut into 4-µm sections and mounted on glue-coated slides. A modification of the avidin-biotin-peroxidase complex immunohistochemical method was performed. Various primary antibodies [human caspase-3 and eukaryotic initiating factor 4E (eIF4E); and rat Ki67] were applied. The expression of eIF4E was assessed using the product of two scores (intensity×% positive), whereas the expression of caspase-3 was represented as positively stained cells per HPF. The Ki67 labeling index was calculated as positively stained cells per 100 cells counted×100%. Histopathological assessment of tumor response was performed by both histomorphological regression analysis (Schneider et al., 2005) and Ki67 labeling index. The extent of histomorphological regression was divided into four categories: grade 1, 75-100% viable residual tumor cells; grade 2, 50-75% viable residual tumor cells; grade 3, 25-50% viable residual tumor cells; and grade 4, 0-25% viable residual tumor cells.

### Thioacetamide-induced cholangiocarcinoma-afflicted rat model

Male Sprague-Dawley rats weighing 180-200 g were housed in a temperature- and humidity-controlled environment with free access to water and rat chow. Cholangiocarcinoma was induced by oral administration of thioacetamide (0.03% w/w in water) for 24 weeks, as previously described (Jan et al., 2004). The treatment regimens were as follows: (1) gemcitabine, 25 mg/kg I.P. every 2 weeks; (2) rad001, 5 mg/kg orally twice every 2 weeks; (3) gemcitabine, 25 mg/kg I.P. every 2 weeks+rad001, 5 mg/kg orally twice every 2 weeks; or (4) saline with the same volume I.P. every 2 weeks for the vehicle group. The animals underwent FDG microPET pretreatment, and at 2 and 4 weeks after treatment, before being sacrificed for histological confirmation.

### ^18^F-FDG microPET imaging to measure metabolic response

Rats had full access to drinking water at all times and were fasted overnight for 8 h before radiotracer injection. A dose of 18.5 MBq (0.5 mCi) ^18^F-FDG was administered and the liver region was scanned for 120 min continuously after intravenous radiotracer injection. Image analysis was carried out at an image analysis workstation (PMOD Technologies, Switzerland). Regions of interest were drawn over the liver tumor and nontumoral liver background. The time-activity curve and tumor-to-liver background (T/L) ratio were calculated. The optimal scanning time point was determined when the highest T/L ratio was reached. SUVs were used to quantify tracer uptake according to the following formula:
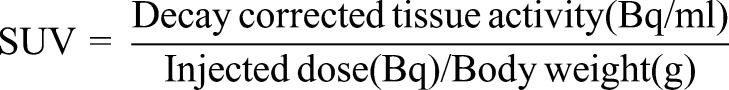
The SUVmax and SUVmean are maximal and mean SUV, respectively, within the voxels of interest (VOIs). Tumor counts and FDG uptake values were obtained in all animals (Yeh et al., 2008).

### Quantification of intratumoral metabolites by NMR

Thioacetamide-induced rat cholangiocarcinoma tumor tissues (∼100 mg) were extracted by dual-phase procedures as previously described (Ackerstaff et al., 2003; Iorio et al., 2005; Venkatesh et al., 2012). Water-soluble extracts were freeze-dried and reconstituted in 700 µl deuterated water (D_2_O, Sigma-Aldrich, USA), and the extracts (500 µl) were then placed in 5 mm NMR tubes. Fifty microliters of 0.75% sodium 3-trimethylsilyl-2,2,3,3-tetradeuteropropionate (TSP) in D_2_O (Sigma-Aldrich) was added to the samples for chemical shift calibration and quantification. ^1^H NMR measurement was performed on a 600 MHz spectrometer (Bruker, Germany): 7500 Hz spectral width, 16,384 time domain points, 128 scans, temperature 298 K, acquisition time ∼5 min. The water resonance was suppressed by a gated irradiation centered on the water frequency. Spectral processing was carried out using the Bruker Topspin-2 software package to quantify the metabolites.

### qPCR for human *FAS*, *CASP8*, *BID*, *APAF1*, *XIAP* and *CASP3*, and rat *Glut-1*, *Hk2*, *Hif1a* and *Vegf*

Total RNA (2 µg) was treated with DNAse 1 (Invitrogen, USA) in a 50 µl reaction mixture. TaqMan primers and probes were used for quantitative detection of human apoptotic genes (*FAS*, *CASP8*, *BID*, *APAF1*, *XIAP* and *CASP3*). Rat *Glut-1* (ABI assay ID, Rn01417099), *Hk2* (Rn00562457), *Hif1a* (Rn00577560), *Vegf* (Rn01511601) and *Gapdh* (Rn99999916) were designed with Primer Express (ABI/Perkin Elmer) using the GenBank accession number. cDNA samples were mixed with 2× Universal TaqMan buffer containing the *Taq* enzyme, primers and probes to a total volume of 25 µl. The thermal cycle conditions were 50°C for 2 min, 95°C for 10 min, and 42 cycles of 95°C for 15 s and 60°C for 1 min. All PCRs and analyses were performed using an ABI PRISM 7700 Sequence Detection System (Applied Biosystems, USA). All samples were run in triplicate.

### Statistical analysis

All continuous variables are expressed as the mean (s.d.). Statistical analysis for continuous variables was performed using Student's *t*-test or one-way ANOVA test where appropriate. Intergroup comparisons of CK were otherwise performed using a box plot, where the data were expressed as the median value. A *P*-value <0.05 was considered statistically significant.

This article is part of a special subject collection ‘Cancer Metabolism: models, mechanisms and targets’, which was launched in a dedicated issue guest edited by Almut Schulze and Mariia Yuneva. See related articles in this collection at http://dmm.biologists.org/collection/cancermetabolism.
